# Dynamic Digital Twin: Diagnosis, Treatment, Prediction, and Prevention of Disease During the Life Course

**DOI:** 10.2196/35675

**Published:** 2022-09-14

**Authors:** Skander Tahar Mulder, Amir-Houshang Omidvari, Anja J Rueten-Budde, Pei-Hua Huang, Ki-Hun Kim, Babette Bais, Melek Rousian, Rihan Hai, Can Akgun, Jeanine Roeters van Lennep, Sten Willemsen, Peter R Rijnbeek, David MJ Tax, Marcel Reinders, Eric Boersma, Dimitris Rizopoulos, Valentijn Visch, Régine Steegers-Theunissen

**Affiliations:** 1 Pattern Recognition Lab Mathematics and Computer Science Technical University Delft Delft Netherlands; 2 Department of Cardiology Erasmus Medical Center Rotterdam Netherlands; 3 Department of Public Health Erasmus Medical Center Rotterdam Netherlands; 4 Department of Biostatistics Erasmus Medical Center Rotterdam Netherlands; 5 Department of Medical Ethics and Philosophy Erasmus Medical Center Rotterdam Netherlands; 6 Department of Industrial Engineering Pusan National University Busan Republic of Korea; 7 Obstetrics and Gynaecology Erasmus Medical Center Rotterdam Netherlands; 8 Web Information Systems Group Mathematics and Computer Science Technical University of Delft Delft Netherlands; 9 Bioelectronics Section, Department of Microelectronics Faculty of Electrical Engineering Technical University Delft Delft Netherlands; 10 Department of Internal Medicine Erasmus Medical Center Rotterdam Netherlands; 11 Department of Medical Informatics Erasmus Medical Center Rotterdam Netherlands; 12 Industrial Design Mathematics and Computer Science Technical University Delft Delft Netherlands

**Keywords:** digital health, digital twin, machine learning, artifical intelligence, obstetrics, cardiovascular, disease, health

## Abstract

A digital twin (DT), originally defined as a virtual representation of a physical asset, system, or process, is a new concept in health care. A DT in health care is not a single technology but a domain-adapted multimodal modeling approach incorporating the acquisition, management, analysis, prediction, and interpretation of data, aiming to improve medical decision-making. However, there are many challenges and barriers that must be overcome before a DT can be used in health care. In this viewpoint paper, we build on the current literature, address these challenges, and describe a dynamic DT in health care for optimizing individual patient health care journeys, specifically for women at risk for cardiovascular complications in the preconception and pregnancy periods and across the life course. We describe how we can commit multiple domains to developing this DT. With our cross-domain definition of the DT, we aim to define future goals, trade-offs, and methods that will guide the development of the dynamic DT and implementation strategies in health care.

## Background

### Overview of the Concept

Interest has been growing worldwide in the virtual representation of a physical asset, process, or system to model and simulate a real-world event. This representation, called a digital twin (DT), can represent the real-time performance or failure incidence of a deterministic system (eg, a factory production line) [1]. Since the introduction of the concept of a DT in 2003, DTs have been developed and used in areas such as construction, power, and oil and gas industries. In the aforementioned industries, the DT served mainly as an umbrella term for managing data and models of a closed system, and these models then guided actions taken in the system. However, for health care, DT is a new concept in need of a working definition. The DT in health care is not a single technology but a domain-adapted multimodal modeling approach incorporating methodologies for the acquisition, management, analysis, prediction, and interpretation of health-related data, aiming to improve medical decision-making and patient lifestyle choices.

Health care providers strive to obtain and use all relevant information on patients for personalized decision-making in clinical practice, considering the available evidence, clinical guidelines, and patient preferences. For example, wearable technologies such as smartwatches have enabled individuals to record their health data continuously, which can form a part of personal health records [2]. They can be very useful for clinical decision-making in practice, and they are currently in use for detection or monitoring of some disorders like atrial fibrillation; however, they are not yet completely in use in practice [3]. Unsolved challenges such as human information overload [4]; variable quality of routinely collected data from medical, lifestyle, mental, societal, and environmental sources; and limited interoperability of digital systems in health care are barriers to use these scattered large data sets, also referred to as Big Data. In addition, the current methodological approaches in evidence-based medicine are not able to use all this information for medical decision-making, as the population generating these data is heterogeneous, and previously discovered relationships between predictor and outcomes might not always hold for subpopulations. To alleviate these limitations, the underlying methods and workflow for data use need to be adapted. Furthermore, currently available analytical models in health care, such as decision aids using risk prediction models (eg, the U-prevent software) [5,6], are often developed based on limited data and the defined outcomes of interest of a small number of health care professional. Combining high-quality, scattered data sets for inference by applying a comprehensive multimodal data management approach in health care such as DT is deemed necessary to design applications that allow for diagnosis, treatment, prediction, and prevention of disease. Moreover, successfully implemented DT has the potential to improve health care by optimizing individual health outcomes from the earliest moment in the life course by offering personalized medicine in primary, secondary, and tertiary health care [7].

**Figure 1:**
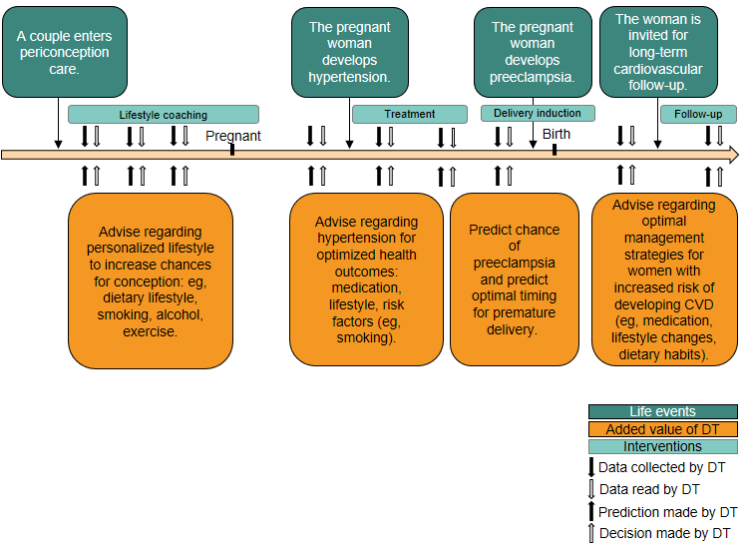
Digital twin in clinical practice is modeled for hypertension starting from the periconception period until later stages of life. There are four discrete steps in which the digital twin can bring additional value by using patient data to recommend interventions optimized for the relevant values and outcomes of interest. CVD: cardiovascular disease; DT: digital twin.

**Figure 2:**
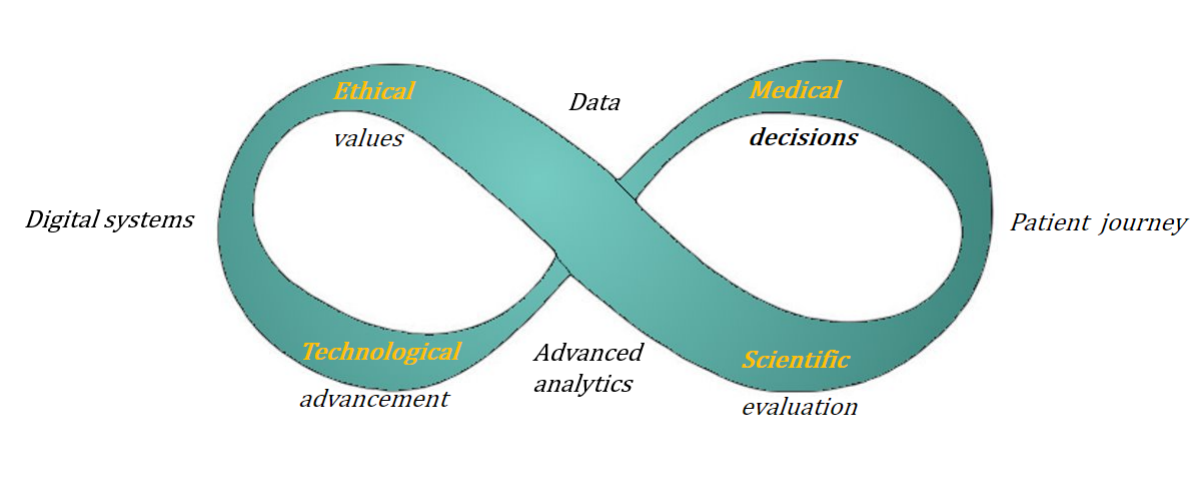
Digital systems and the patient health journey are improved by a continuous feedback loop across domains interacting to develop a digital twin.

Since 2015, publication of papers describing the concept of applying a DT to health care to solve health challenges (eg, reducing adverse outcomes in certain patient populations or understanding important factors such as dietary factors or biomarkers) [8-13] has increased. However, literature descriptions indicate open theoretical and practical challenges that need to be addressed before we can work toward efficiently implementing a DT in health care. Challenges are related to (1) redefining a target population and matching data set to develop a DT or (2) implementing a physical or data-driven approach with limited data and follow-up to learn causal personal patterns. In addition, we suggest a convergence of experts in the medical, technical, scientific, and ethical domains, which is required to design a DT that meets requirements from several theoretical backgrounds. In our vision, DT development starts with the identification and description of a medical problem such as the prediction of cardiovascular complications in a patient who experienced hypertension during periconception (Figure 1). Solving such a medical problem involves striving for the best health- and value-based outcomes and addressing ethical value goals such as health equality (Figure 2). With these outcomes and value goals in place, a technological system incorporating best methodological practices such as reliability and reproducibility can be developed. After the initial system is developed, many feedback loops between domains occur in order to optimize requirements across domains. This digital health system is then studied to develop best practices using the scientific domain and keeping the healthy patient life course journey in mind. In an open system such as the life course, as opposed to a closed physical system, causal drivers for change in health conditions might not be known; therefore, the DT uses algorithms that learn drivers of dynamic data such as user experiences, medical data fields, medical scans, etc. To serve that purpose, the DT acquires time-series data and updates predictions in an online and data-driven manner. Additional challenges are defining medical applications of a DT in health care and considering ethical values.

In this viewpoint paper, we address the aforementioned challenges and put forward a vision for a dynamic DT in health care for optimizing individual patient health journeys characterized by healthy outcomes and positive experiences. Our cross-domain, which has the ethical, medical, technical, and scientific definition of the DT, will define future goals, trade-offs, and methods to guide the implementation strategies and iterative development of a dynamic DT in health care. In our paper, we propose a dynamic DT for health care that applies to the management of dynamic patient data and models. These models of health and disease are dynamic because they are developed, trained, tested, and updated to meet the dynamic value goals stemming from ethical, medical, and technological domains. Additionally, the effect of these interventions (such as personal lifestyle advice) guided by models is scientifically evaluated and updated to continually strive for better health outcomes (Figure 2).

### Potential Future Application of a DT in Health Care

Currently, the translation from data to evidence relating to disease ontology, causation, and effectiveness of treatments into clinical practice is a slow and partially data-driven process [14], and even for some of the recommendations in the clinical practice guidelines, there is no robust evidence available [15]. The dynamic DT can support a health journey by providing easy access to comprehensive patient data for the patient and health care providers. The DT can integrate data from health care sources like hospitals, general practitioners, and laboratories and from home health monitoring devices such as wearables. Real-time integrated access to data will allow analysis on dynamically acquired data. This feature enables health care professionals to get a relevant view of factors influencing disease and health conditions of a patient, which can guide actions to optimize health care pathways. Different prediction algorithms can be used to answer medical questions, and real-world personalized predictions can be updated to meet the dynamic circumstances of individuals and their medical history. Testing these prediction models in the real world prospectively can help to define and research hypotheses about potential causal relationships between prediction algorithms and outcomes [16]. Formal testing and evaluating medical decisions in a DT can result in changing the focus of scientific studies to incorporate testing the implementation of a DT-powered intervention such as patient-tailored lifestyle advice in the absence of a data-driven decision framework. Therefore, this DT approach can expedite the process of knowledge translation into medical decision-making in clinical practice while striving for iterative improvement of elements in different domains of the DT.

### Example of a Patient Journey From the Earliest Moment of the Life Course

The periconceptional period refers to the 6 months around conception; it is the earliest and one of the most critical periods in life, with long-lasting impact on health and disease later in life and in future generations [17]. The patient journey and data generated during this period can help to illustrate our vision of the dynamic DT and how it would intervene in medical practice. To develop a DT from the earliest moment of the life course, the first set of data would include static (demographics) and dynamic (conditions such as subfertility, hypertension, lifestyle, and vital information from wearables) covariates relating to the parents. This DT supports dynamic medical decision-making (Figure 1). The first example is based on subfertility, for which enrollment in the evidence-based lifestyle coaching program is recommended. This eHealth coaching is not static but dynamic as it gives advice based on the variable input of the individual. For example, if healthy food intake is sufficient, parents will be empowered to maintain this behavior. A second example of how the DT can be used is aiding physicians to prevent short-term adverse outcomes of preeclampsia in pregnant women. The mobile phone app, a smartwatch, and lab measurements combined can yield predictions of risk before and after intervention, empowering medical decision-making and resulting in a better outcome for mother and baby. A third example to highlight the long-term benefits of a DT is hypertension. By mechanisms not fully understood, high blood pressure and preeclampsia during pregnancy yield increased cardiovascular risk for the unborn child and mother in later life years [18-20]. This data-driven association can make more comprehensive follow-up of these individuals valuable as this information can be used to monitor and potentially prevent worse outcomes such as cardiovascular disease. In our example, the DT has enacted medical decision rules driven by pattern recognition in patient data. In general terms, the rules obtained from pattern recognition systems are chosen so that they optimize for healthier outcomes.

### Why Now: Innovations That Power the DT

In 2016, a pivotal paper was published that described how data should be managed according to the following 4 principles: findable, accessible, interoperable, and reusable (FAIR) [21]. These principles promote data accessibility to power innovation and are the basis for the following 4 technical innovations that accelerate the progression of DT in health care. The first innovation is data storage, where we can securely store big data in the cloud by designing access rights for each service and algorithm [22]. Data collection innovations such as wearables, which can act as continuous vital sign monitoring systems, feed the new data to the storage keeping the data up to date and relevant. Connectivity innovations such as the Internet of Things then trigger the training and prediction algorithms with the updated information. Finally, computing innovations such as more powerful data processors like tensor processing units can fit bigger and more flexible models with higher performance to yield better representations of disease. These innovations have changed the way we form hypotheses about the physical reality. Increased data and processing power formats require and allow for new dynamic pattern recognition methods that can be described as computer pattern recognition or artificial intelligence [23].

These innovations power the DTs and allow for deep personalized predictions that leverage patient-specific dynamics such a specific disease pathway relating to biomarker panels based on genomics, metabolomics, or proteomics, which have previously been prioritized or identified. These predictions need to be translated in a concise way to an individual, patient or health care provider. For example, a score, dashboard, or written advice can guide individuals to healthy behavior. In our example, continuously monitored medical and lifestyle data of a couple contemplating pregnancy supplies the DT with data that can be used to give personalized integrated medical advice and brings personalized medicine closer [24].

### Themes in Pattern Recognition

There are multiple paradigms for the underlying data analytic platform of a DT relating to pattern recognition approaches. We can use a data-driven or physics-based approach. Physics-based models are based on the understanding of the phenomenon and formulation in a mathematical model, with underlying assumptions that potentially oversimplify the phenomenon [25]. Data-driven (associative) models avoid these assumptions, but they lack interpretability and are sensitive to bias in the model development data. We should choose our (inductive) bias in a way that allows us to optimize interpretability and predictive performance by leveraging domain medical expert–level knowledge. When the physical drivers of disease are not known, these associative data-driven methods can generally be used to diagnose patients, but they cannot identify causation. An alternative approach within the associative methodology is to enrich models with counterfactuals, which leverage counterfactual information to causally weight predictions [26].

In the DT for health care, pattern recognition algorithms are used for predicting continuous outcomes such as blood pressure, classification of diseases, and risk assessment. Medical data generally comprise different types of data and algorithms. For example, they include longitudinal data with missing predictors and variables, which can be used to enrich the model. Research on how to associatively predict with longitudinal data, population-level data, and data with missing values is ongoing. A statistical modeling approach, which can relate a limited number of predictors to a longitudinal outcome with missing values, uses linear mixed models [27]. Deep-learning methods [28,29], where we flexibly fit either a neural network architecture or many random kernels, are used when a large number of predictors is present. This modeling approach seems to perform well in this domain, but least absolute shrinkage and selection operator regression-based techniques may also yield good performance on this type of data [30].

Medical patient data can comprise higher dimensional data, such as echography, and multiple sensor data, such as electrocardiograms, which are continuously collected in a setting such as the coronary care unit. Imaging data is typically only acquired once and not collected continuously. Although each modality may be information rich, the number of measurements per individual may be limited, causing models to overfit on that individual. To prevent overfitting, we use feature selection and methods that can find a lower representation of these data, such as an auto encoder. Feature selection and engineering can prevent overfitting and summarize high-dimensional data in a feature vector, which can be used to describe disease progression.

As models are implemented in clinical practice, other challenges, such as model drift and the appearance of new class definitions, can arise that cause previously found associations to change. The online learning paradigm can adapt to this change by continuously updating the model with new information and thereby learning to adapt to changes in the environment [31].

Last, because the medical profession generally deals with interventions, another theme of interest is causal machine learning, where the focus is on creating a model that can predict the change in target output if a predictor were changed (eg, change in diet or blood pressure). The challenging part is inferring causality from observational data, as in medicine we would not only like to associatively predict but intervene in patient health journeys to prevent adverse outcomes. The current gold standard is randomized intervention data, but these data are resource-intensive to collect, and the clinical trial setting does not reflect real life, so the distribution of these data may not fit the general population [32]. However, there are powerful alternatives for causal questioning that may have a future in drug efficacy and safety evaluation on real-world data [33]. Moreover, there are tools being used for evaluating interventions with methods, which can possibly extend into other medical domains [34].

In summary, applying accurate and understandable models on data that fit well to the target individual will be paramount to the success of the DT. Equally important is deconvoluting the causal factors that influence health outcomes, as this knowledge will power interventions based on personalized causal factors. Using a combination of these methods can form the technical analysis part of the DT framework (Figure 2).

### Dynamic Digital Wins

**Figure 3:**
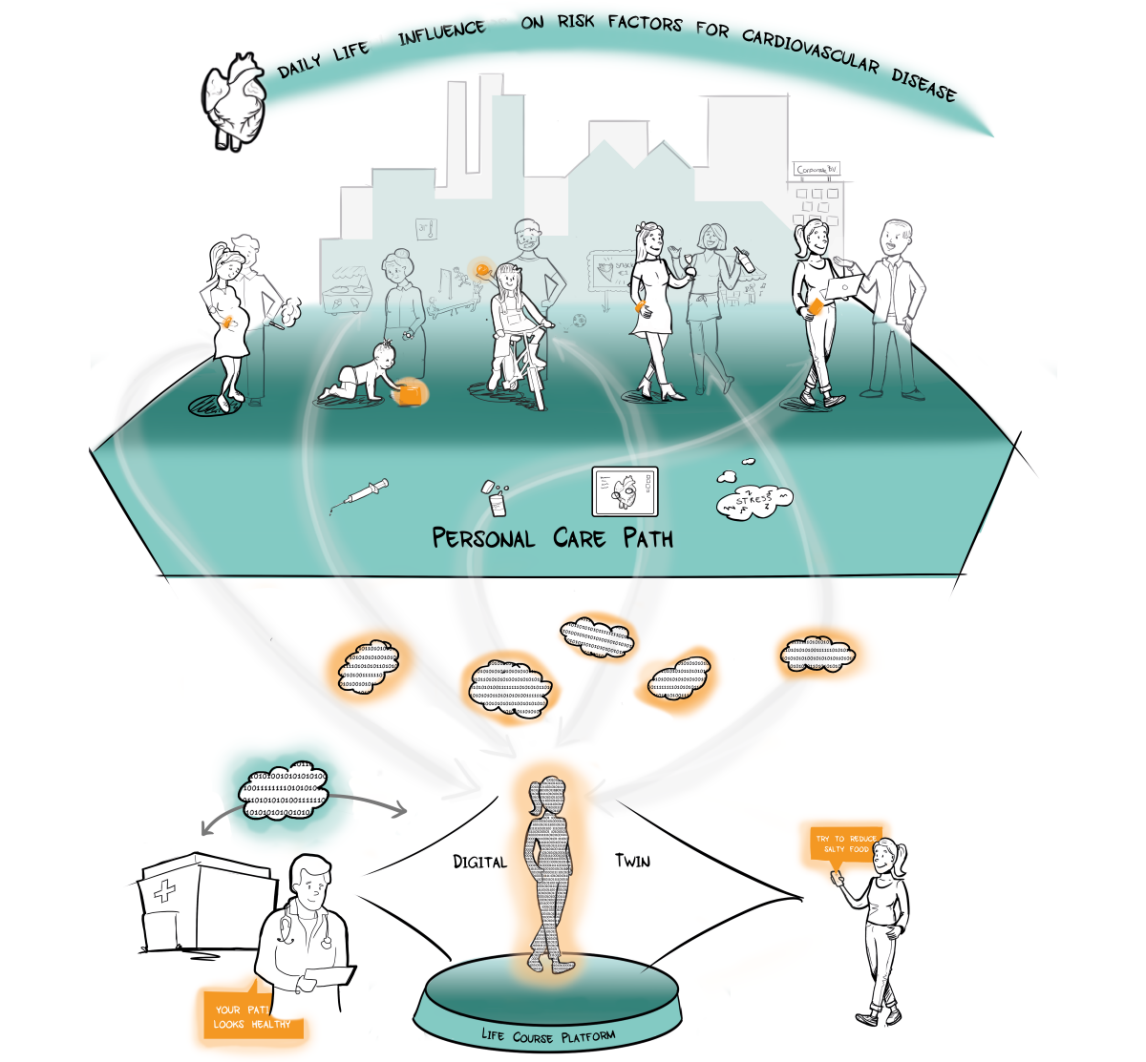
A digital twin encompassing different aspects of the life course can be queried to improve medical decision-making to reduce cardiovascular complications. In orange, we show data (and data generators). In green, we display the digital life course platform and real-life course. Together they supply information to each other to support a healthy patient lifestyle by defining a personalized care path.

A dynamic DT (Figure 3) is a twin whose relationships—and the data contained within—change over time as the conditions of people’s lives change over time (eg, a young healthy individual who needs preventive care to increase the chance of fertility, a patient treated for a life-threatening disease at the coronary care unit). The dynamic aspect also refers to the changing of the model targets according to the relevant question and value trade-off. As an individual experiences different phases of life, the relevant dynamic DTs are triggered to make a prediction using the up-to-date data. To keep the predictions relevant to the patient, the DT needs to be updated in a continuous manner, preferably with the least amount of burden on the individual. The continuous monitoring of vital signs (for patients in a hospital setting and at home) using wearable devices allows for prompt detection of developing pathologies and early intervention, which may not be possible using standard intermittent vital sign measurements [35]. To have a successful implementation of a dynamic DT, a minimal requirement is to start with a FAIR data set. On this data set, algorithms must be trained and tested, and this output needs to be translated into medical or lifestyle advice. The next step is the validation of this advice to see if this decision-support tool improves patient outcomes. For the full DT, there are multiple requirements: (1) continuous collection of, for example, medical and user experience data, (2) integration of new data with existing data in the databases, and (3) improvement of accessibility of systems that access and store new data [36].

### Data Management

The first requirement in data management is the collection of high-quality data, which includes similar distribution of patients and individuals as the target individual. As the clinical data is collected from different sources, an important challenge is to synergize, homogenize, integrate, and standardize the heterogeneous data to have a standardized data model. In such a data model, we transform the local data source into the common data model via extract, transform, and load procedures [37]. This data model should live in a system that can sustain FAIR principles. Moreover, patients/researchers and health care providers should have an easy and flexible way to add or remove medical and lifestyle data sources [24]. Data security and privacy are important for DT development in the medicine and health care sectors. Open challenges are cleaning and preparing the data [38] and protecting the privacy of data residing on cloud-based data storage platforms [39]. The rising popularity of storing health data on cloud platforms requires deliberate design in managing data access control and data ownership in combination with schemes such as federated analysis.

### Values

Values are the quality of a digital health service as it is experienced by an individual in relation to their needs [40]. Based on this definition, values are created when users such as patients, clinicians, nurses and others use the DT to address their challenges [41,42]. Representative examples of such challenges were identified by the World Health Organization in 2018 [43] and include efficiency, cost, and access to information.

In our early life course patient journey example, the DT can solve information challenges by collecting high-quality data through wearable devices and then providing easy access and visual summaries of the data to the patient and their treating health care professional. Additionally, the DT can provide personalized predicted probabilities of adverse health outcomes and suggest treatment consisting of lifestyle advice or drug treatment to lower those probabilities. The DT can aid the use challenges of low adherence to treatment by visualizing the effect that treatment may have on future health outcomes so that the patient can better understand and be motivated by the potential benefit of adherence to treatment. The DT addresses efficiency challenges by dynamically updating a patient’s predicted probabilities for adverse health outcomes, which enables an early reaction to increased risks. This information may also be used to choose the optimal time between health checks and interventions; in limited care situations, it may be used to prioritize patients with greater needs in triage settings. The cost challenges can be addressed by early risk detection and interventions of the DT, which may prevent the need for costly treatments at later stages of disease progression. To create a valuable DT for users, it may help to include lead users in the development team [24].

### Legal and Ethical Considerations

#### Value Trade-offs

The DT allows us to optimize for healthy outcomes at the cost of other values (eg, fairness). While incorporating more health-related data might increase a prediction’s accuracy, it could also raise concerns about surveillance health care and erode the trust between the health care system and individuals [44]. A DT measuring patient data (such as sleep) and the feedback of health guidance could even affect the parameters it is measuring. For example, commercial consumer wearables are not accurate in estimating sleep [45], and the results may worry consumers and even cause sleep problems. An increased number of measurements may identify patients that are considered atypical. Currently, we lack a clear understanding of how to interpret slight deviations from the normal ranges and means and whether these asymptomatic deviations will lead to future diseases. Balancing the ideal of early diagnosis and reducing overdiagnosis and overtreatment can therefore be challenging [46,47]. The attempt to introduce personalized lifestyle advice to curb lifestyle diseases such as obesity, diabetes, and hypertension also risks promoting an overly individualized view of health management.

Additionally, many have expressed concern about worsening existing health inequity [48,49]. People from lower socioeconomic backgrounds might not have the means to access the digital device required for a DT service, or they might not know how to benefit from the information provided to them due to cognitive constraints such as limited digital or health literacy and external constraints. Instead of empowering users by offering them more health-related information, the DT might burden users with a sense of guilt or anxiety and give rise to the idea that users who do not make the advised change could be accused of being responsible for their adverse health outcomes [50-52].

#### Data Governance and Accountability

The digitalization of health care gives rise to new legal and accountability issues. For instance, who owns the health data gathered by the DT? Is it owned by the patient, the health care provider, or a third party? Is it morally permissible for a DT service provider to sell user data to a third party? To avoid data being used against the patients’ best interest, Schwartz et al [24] suggest 3 principles: (1) patients own their data, which empowers patients to protect the privacy of their data against misuse, (2) patients must provide explicit informed consent for the use of their data, and (3) advocacy efforts should enshrine patient data ownership and access into law. The implementation of these principles poses technical and legislative challenges. Additionally, DTs are susceptible to biases present in the data from which they are developed. Data included in the DT should be reviewed and methods to remedy biases should be considered to avoid the perpetuation of historical biases [24]. De Laat [53] and Nissenbaum [54] describe the obstacles of accountability of machine learning algorithms and how this relates to shared development, human and computer errors, and a culture where it easy to blame the technology. There is also a lack of understanding of how algorithms work [53,54]. Transparency of decision rules and oversight of the decision-making algorithms by governing bodies could solve these outstanding obstacles.

### Scientific Domain

In this paper, the DT was defined in terms of requirements stemming from medical, technological, and ethical domains, but to evaluate the effectiveness of such a DT system, we require scientific convergence to test its merit and limitations (Textbox 1). In translational medical science, we aim to generate knowledge in clinical practice so we can intervene and improve health care processes [55]. To achieve this aim, we need to understand the disease process and uncover the relevant causal pathways that influence health outcomes. DTs can be helpful in combining cross-domain knowledge. As a DT incorporates more than one data set, such as physiological measurements, questionnaires, and lifestyle factors, there are many rich features for each patient or individual ready to be used for pattern recognition. Knowledge about individual contributors of disease progression, in turn, could lead to the identification of subtypes of disease with different disease ontologies and treatments, described as deep phenotyping [13]. The DT platform may allow us to test our predictions more efficiently in an umbrella or real-world trial [16,56]. Predicting patients prospectively and monitoring for outcomes may allow us to test the accuracy of the predictions from the DT. In a second step, randomized intervention based on these predictions can unveil causal factors, and this in turn can help us test our scientific hypotheses using real-world evidence and thereby moving science forward by generating medical knowledge and developing novel technical methodologies.

**Benefits of digital twin development in health care.**

Health agency and promoting healthy outcomes

- More accurate diagnosis using integrated data
- Improved treatment selection for patients
- Prognosis of patient disease trajectories
- Real-time remote monitoring of health state
- Simulation of treatment and care processes to guide policy

Fair data and validation of research

- Data structures in the digital twin will make data more accessible
- Validation of scientific results provide an implementation framework for decision-making

Algorithm development for medical decision-making, diagnosis, and prevention

- Innovation in algorithms allows us to make more accurate predictions on unseen data
- Causality is inferred from observational data
- Privacy is preserved during data sharing and analysis
- Uncertainty is quantified from different sources and considered during the decision-making processes

## Conclusion

In conclusion, translational science and medical care can be improved by following a DT life course approach: high-dimensional data collection and storage, patient trajectory modeling, outcome predictions, testing, model interpretation, and implementation in clinical practice. By committing multiple nonmedical domains to developing a DT, we aim to improve patient care journeys in a systematic and diligent way.

## Future Research Opportunities

Our vision of a dynamic DT allows for collaboration of many researchers from different domains, where we can align our research applications in order to develop a dynamic DT. Some subjects related to the DT have not been fully investigated, and new opportunities for research have been defined. A part of the technical domain, which is under active investigation, is how we can leverage personal and lifestyle factors to reach more accurate predictions on unseen data. This involves but is not limited to using nonrandom and sparse sampling as a predictor matrix to improve our models and infer causality.

We also need more work on how to identify new classes, such as disease types based on new distance metrics, and how to tune our models to meet patient preferences and different privacy settings. A large body of research in the ethical domain is dedicated to value trade-offs in the DT setting and investigates how we can balance values such as fairness, equality, and health. We are also concerned with data storage and federated data analytics, and we performed analysis on harmonized big data sets. Optimal design of the DT application and user interaction is required to meet the requirements of users while optimizing for values arising from different domains. The science domain should come into play to judiciously evaluate the data, models, application, and ability of the technologies to impact patient life course trajectories and decrease the incidence of disease.

## Abbreviations

DT: digital twin
FAIR: findable, accessible, interoperable, and reusable
